# Neural Changes in Borderline Personality Disorder After Dialectical Behavior Therapy–A Review

**DOI:** 10.3389/fpsyt.2021.772081

**Published:** 2021-12-17

**Authors:** Adam Iskric, Emily Barkley-Levenson

**Affiliations:** Department of Psychology, Hofstra University, Hempstead, NY, United States

**Keywords:** borderline personality disorder, dialectical behavior therapy (DBT), neurobiology, magnetic resonance imaging (MRI), near-infrared reflectance spectra (NIRS)

## Abstract

The biological component of the biosocial theory of emotion regulation stipulates that borderline personality disorder (BPD) arises from biological vulnerabilities to heightened emotional reactivity. Comprehensive reviews have consistently implicated abnormalities in the amygdala, anterior cingulate cortex, and hippocampus in the neurobiology of BPD. While Dialectical Behavior Therapy (DBT) is the leading evidence-based psychotherapy for the treatment of BPD, there remains a paucity of literature examining changes in the neurobiology of BPD following DBT treatment. Nine studies were identified that examined neurobiological changes in BPD after the completion of DBT. Results indicated that there was significant deactivation of amygdala activity as well as the anterior cingulate cortex in patients with BPD after DBT treatment. As well, several studies found after DBT treatment, BPD patients had a decreased activity in the inferior frontal gyrus in response to arousing stimuli and increased activity in response to inhibitory control. Future research on the neurobiological change after DBT treatment can help clarify biological mechanisms of change in BPD.

## Introduction

Borderline Personality Disorder (BPD) is a debilitating mental illness characterized by emotion dysregulation, poor impulse control, identity disturbances, recurrent suicidal or self-harming behaviors, and unstable interpersonal relationships (1, 2). The etiological origins of BPD have been conceptualized within the biosocial theory of emotion dysregulation, which theorizes that BPD arises from a combination of both (a) biological vulnerabilities to heightened emotional responsivity as well as (b) a social environment that involves the invalidation of emotional expression and the reinforcement of emotional arousal (3). This review article will primarily focus on the biological components of BPD.

Comprehensive reviews have consistently demonstrated the multifaceted neurobiology of BPD in adults (2, 4, 5) as well as adolescents (6), along with the neurobiology of non-suicidal self-injury, a prominent and pervasive symptom among individuals with BPD (7). For example, abnormalities in the amygdala and anterior cingulate cortex (ACC) have been consistently implicated in the neurobiology of both BPD as well as individuals who engage in non-suicidal self-injury (2, 5–7). More specifically, a meta-analysis of neuroimaging studies on emotion processing showed left amygdala hyperactivity and reduced dorsolateral prefrontal cortex (dlPFC) among individuals with BPD (8, 9). Moreover, abnormalities in the hippocampus have been linked with BPD in adults (2, 5).

Dialectical Behavior Therapy (DBT) is the leading evidence-based psychotherapy for BPD that emphasizes the role of emotion regulation and targets the acquisition of skills and techniques to encourage control over maladaptive behavioral patterns (10–15). However, despite the plethora of research on the neurobiology of BPD, there have only been a handful of studies examining *changes* in neurobiology following DBT treatment of BPD. The purpose of this article will be to review the literature on how DBT can influence the neurobiology of BPD.

## Methods

A review of the current literature was carried out by searching for articles in Pubmed/Medline, PsycINFO, and Google Scholar, along with articles obtained from the references of other articles. The reference sections of the review articles mentioned above [i.e., (2, 4–7)] were also examined to determine if there were any other studies that examined the neural substrates of BPD after treatment. Keywords used for this review included “dialectical,” “borderline,” “brain,” and “neural.”

## Results

A total of nine studies were identified for the review. Seven studies used functional magnetic resonance imaging (fMRI) and two used functional near-infrared spectroscopy (fNIRS). Within the nine studies, four of the fMRI studies (8, 16–18) and two of the fNIRS studies (19, 20) were obtained from the same data collection. No studies using positron emission tomography (PET), magnetoencephalography (MEG), electroencephalogram (EEG), or magnetic resonance spectroscopy (MRS) were identified. Table 1 describes the characteristics of each study.

**Table 1:** Summary of the reviewed studies.

**References**	**Participants**	**Imaging method**	**Type and duration of DBT program**
Schnell and Herpertz (21)	6 female BPD vs. 6 HCs	fMRI	12-week inpatient program
Goodman et al. (11)	11 BPD vs. gender-matched HCs	fMRI	12-month outpatient program
Schmitt et al. (17)	32 female BPD in DBT vs. 16 female BPD in TAU vs. 24 female HCs	fMRI	12-week inpatient program
Winter et al. (18)	31 female BPD in DBT vs. 15 female BPD in TAU vs. 22 female HCs	fMRI	12-week inpatient program
Niedtfeld et al. (8)	28 female BPD in DBT vs. 15 female BPD in TAU vs. 23 female HCs	fMRI	12-week inpatient program
Mancke et al. (16)	31 female BPD in DBT vs. 17 female BPD in TAU vs. 24 female HCs		12-week inpatient program
Farrés et al. (22)	10 BPD in DBT mindfulness group vs. 14 BPD in DBT interpersonal effectiveness group	fMRI	10-week outpatient program
Rodrigo (19)	18 BPD with self-harm	fNIRS	6-months out of a 1-year outpatient DBT program
Ruocco et al. (20)	18 BPD with self-harm	fNIRS	6-months out of a 1-year outpatient DBT program

### FMRI Findings

Several studies focused on neural changes observed using fMRI resulting from 12-week DBT treatment programs for individuals with BPD. Schnell and Herpertz (21) examined whether the increased regulation of affective arousal after a 12-week in-patient treatment program would translate into changes in the specific brain regions involved in affect regulation. Five longitudinal fMRI scans were completed, wherein participants viewed a set of images designed to induce emotional arousal. Patients with BPD who received DBT treatment displayed a significant reduction in activation in the right caudal anterior and posterior cingulate cortices, the right middle temporal gyrus, and the left anterior insula in response to the arousing stimuli, while control patients displayed no significant reduction among the same brain regions. Moreover, the subgroup of therapy responders (i.e., four of the six patients with BPD) were the only patients who also exhibited decreased activity in response to the arousing stimuli in the right inferior and medial frontal gyrus, the left amygdala, and the bilateral hippocampus. One strength of this study was the relatively large number of repetitive scans used to counter the criticism of typical pre-post designs–one round of neuroimaging before treatment and the other afterward–which may reflect general symptom improvement rather than the specific effects of psychotherapy (23).

Another study compared patients with BPD with gender-matched healthy controls (HC) with no Axis I or II disorders or family history of an Axis I disorder using a 12-month DBT program (11). During both the pre- and post-treatment fMRI scans, all participants viewed an intermixed series of unpleasant, neutral, and pleasant pictures. Results indicated that while the BPD group exhibited an overall decrease in amygdala activation post-treatment, the HC group showed overall amygdala activation that was similar at baseline and 12 months. Furthermore, compared with healthy controls, patients with BPD showed a pattern of greater decrease from pre- to post-treatment in amygdala activity for all three pictures types, but particularly in the left hemisphere and during the repeated emotional picture conditions (unpleasant and pleasant). Finally, among the patients with BPD, reduction in amygdala activity to repeated unpleasant pictures (i.e., improved amygdala activation) following DBT was associated with improved emotion regulation. One of the strengths of this particular study was that all BPD participants were unmedicated, so this study was able to remove the potential influence of medication on positive treatment outcomes or specific brain changes (11).

Four separate studies were part of a larger clinical trial on alterations in neural correlates of emotion regulation in BPD after DBT (8, 16–18). The first study examined the impact of a 12-week DBT program in women with BPD compared to women with BPD in the treatment-as-usual (TAU) and female healthy controls with no current or past psychiatric diagnoses (17). During the pre- and post-treatment fMRI scans, the women with BPD in the 12-week DBT program were asked to view either a negative or a neutral picture and asked to either (a) reappraise their emotion by imagining that the situation was not real or that they were a detached observer (*reappraisal condition*) or (b) view the pictures attentively without trying to alter their affective reaction (*maintain condition*). Results indicated that the BPD patients in the DBT group experienced a stronger activity decline in the anterior insula and dorsal ACC post-treatment compared to healthy controls, while there was no significant difference between activation of the orbitofrontal cortex (OFC) and the dlPFC between groups. Furthermore, in DBT patients, the decline of anterior insula activity during reappraisal (but not passive viewing) of negative pictures was significantly correlated with a reduction in BPD symptom severity. Moreover, BPD patients experienced increased anterior cingulate connectivity to medial and superior frontal gyrus, superior temporal gyrus, and inferior parietal cortices after DBT. Finally, DBT responders demonstrated diminished activity post-treatment in the right amygdala, subgenual, perigenual and dorsal ACCs, the medial and left OFCs, and right dlPFC during reappraisal compared to DBT non-responders. DBT responders also showed increased connectivity from the dlPFC to the OFC, superior temporal gyrus, posterior insula, cingulate gyrus, and thalamus over time compared to DBT non-responders (17).

The second study compared female patients with BPD undergoing 12 weeks of DBT with female patients with BPD who were not enrolled in DBT and female healthy controls (HC) with no current or lifetime psychiatric disorder and no psychotropic medication (18). Participants were instructed to either (a) passively view (i.e., *passivity condition*) or (b) memorize letters (i.e., *memorization condition*) before being confronted with negative or neutral pictures in a distraction task during the pre- and post-treatment fMRI scans. After the presentation of the pictures, participants had to indicate as fast as possible when they (a) saw the letter “o” or (b) whether the letter presented was in the memorized string of letters. The *memorization-negative* condition was used as a way to measure distraction as an effective emotion regulation strategy in reducing negative affect, particularly when stimuli are highly arousing (24). Results indicated that patients with BPD who received DBT showed decreased activation in the right inferior parietal lobe and supramarginal gyrus during distraction from negative rather than neutral stimuli when compared to both controls. This decrease in activity was also significantly correlated with improvement in borderline symptom severity. Finally, DBT responders exhibited decreased right perigenual anterior cingulate activity when viewing negative (rather than neutral) pictures. The authors suggested that the results emphasize that specific neural changes are associated with distraction from negative stimuli after the completion of DBT among BPT patients (18).

The third study (8) examined 12-week DBT treatment among female patients with BPD with affective instability and NSSI during the last month compared to female patients with BPD assigned to treatment as usual (BPD + TAU) and female healthy controls (HC) with no lifetime psychiatric disorders. All patients participated in two fMRI sessions 12 weeks apart (pre- and post-treatment for the BPD + DBT group), viewing negative or neutral picture stimuli coupled with either a baseline temperature thermal stimulation (32°C), or a painful temperature stimulus based on each individual subject's pain threshold, as a means of investigating whether DBT altered pain-mediated affect regulation at the neural level. Results indicated that amygdala deactivation in response to negative pictures combined with painful temperature was observed only in the BPD + DBT group at baseline and was no longer present post-treatment, while the BPD + TAU and the HC groups experienced no significant change in amygdala activation in response to negative pictures and painful temperature. These results suggest that the emotion regulation skills taught within the DBT skills group might have enabled patients to regulate amygdala activity more efficiently, reducing at a neural level the role of pain in coping with negative affect (8).

The fourth study compared female patients with BPD undergoing 12 weeks of DBT with female patients with BPD who were receiving TAU (16). Participants completed two separate fMRI scans at baseline and at 12-week follow-up. Results indicated that patients with BPD receiving DBT showed an increase of gray matter volume in the dorsal and rostral ACC, inferior frontal gyrus, and superior temporal gyrus together with an increase in gray matter volume in the angular gyrus and supramarginal gyrus compared to patients with BPD receiving TAU. DBT treatment response was also significantly correlated with increase of gray matter volume in the angular gyrus. The authors concluded that DBT may significantly alter brain regions that are explicitly involved in cognitive regulation of emotional information and mentalizing (16).

In addition to examining the impact of a full DBT program on various brain structures, research has also been conducted on the specific modules of DBT. One study compared the neural correlates of BPD patients in a 10-week DBT mindfulness (DBT-M) group with BPD patients in a DBT interpersonal effectiveness (DBT-IE) group (22). DBT is primarily composed of four different modules, including mindfulness, interpersonal effectiveness, emotion regulation, and distress tolerance (3). During both pre- and post-fMRI sessions, participants performed a sequential-letter version of the *n*-back task whereby participants had to indicate when the current letter matches the one from *n* steps earlier in the sequence.

Results indicated a significant increased activation of the left anterior insula extending to the frontal inferior operculum after the intervention across both DBT-M and DBT-IE groups (22). Furthermore, BPD patients in the DBT-M group showed higher deactivation in a cluster at the medial occipital location extending bilaterally from the calcarine to the cuneus and superior occipital gyri while performing the *n*-back task compared to the DBT-IE group. However, there was no post-treatment difference in default mode network activation or deactivation across both groups, a brain region associated with various cognitive processes such as introspection, envisioning the future, and self-reflective thought (25). There was also no significant correlation between BPD symptoms or mindfulness outcomes and activation/deactivation of the cluster at the medial occipital location extending bilaterally from the calcarine to the cuneus and superior occipital gyri (22). These particular findings highlight how specific components of DBT, including mindfulness and interpersonal effectiveness, may be uniquely associated with post-treatment changes in specific brain regions.

### Functional Near-Infrared Spectroscopy (FNIRS) Findings

While the majority of DBT research has been conducted using fMRI technology, two studies implemented a functional Near-Infrared Spectroscopy (fNIRS) (19, 20). The first study examined 29 self-harming patients with BPD who completed ~6-months of a standard 1-year outpatient DBT program (19). Out of the 29 initial patients, 18 of them remained in treatment at the 6-month follow-up. During both pre- and post-treatment fNIRS scans, participants completed a go/no go task of response inhibition that required participants to press a button with their right index finger to “go” stimuli (i.e., a green circle) and withhold a response to “no-go” stimuli (i.e., a red circle). Results of this study indicated that after 6 months of DBT, BPD patients demonstrated less activation bilaterally in regions encompassing the middle and inferior frontal gyri as well as more activation in the right medial PFC during inhibitory control. As well, these activation patterns were associated with improvements in BPD symptoms (19).

After the 6 months of DBT, differences in activation during inhibitory control were also observed based on changes in affective, cognitive, impulsivity, and interpersonal domains (19). More specifically, BPD patients with less improvement in affective symptoms had an increased activation in the right medial PFC during inhibitory control. In contrast, BPD patients with more improvement in affective symptoms had higher activation in the lateral PFC at the 6-month follow-up during inhibitory control. As well, both BPD patients with less improvement as well as more improvement in cognitive symptoms showed more activation within the lateral PFC during inhibitory control. BPD patients with more improvement in cognitive symptoms also demonstrated more activation within the right PFC during inhibitory control. Moreover, while BPD patients with less improvement in impulsivity demonstrated higher activation in the left and right medial PFC during inhibitory control, BPD patients with more improvement in impulsivity demonstrated higher activation in the left and lower activation of the right medial PFC during the same task. Finally, while BPD patients with less improvement in the interpersonal domain showed higher activation within the right medial PFC, BPD patients with more improvement in the interpersonal domain demonstrated higher activation in the right PFC (19). A follow-up analysis using the same sample found that reductions in self-harm over the treatment period were associated with increases in activity in right dlPFC even after accounting for improvements in depression, mania, and BPD symptom severity (20).

## Discussion

The main findings of this review suggest neurobiological changes in brain function after the completion of DBT treatment among patients with BPD. Firstly, two studies found a significant deactivation of amygdala activity after the completion of DBT treatment particularly among DBT treatment responders (11, 21). These findings are particularly important, given the involvement of the amygdala in the perception and processing of emotion (11, 26) and the hyperactivity of the amygdala among patients with BPD as well as individuals who engage in non-suicidal self-injury (2, 5–7). Interestingly, one study found a significant deactivation of amygdala activity in response to negative pictures and painful temperature at baseline, but not at post-treatment (8). This finding may suggest that the use of non-suicidal self-injury in response to emotional arousal can reduce amygdala activity among patients with BPD prior to treatment (8).

Secondly, two studies found significant decreases in activation in the ACC after DBT treatment among patients with BPD (17, 21), while two other studies found a significantly reduced activity in the ACC specifically among DBT responders (17, 18). One study also found a significant increase in gray matter volume in the ACC (16), while another study found a positive alteration in functional connectivity between the left amygdala and the dorsal ACC when negative pictures were combined with painful stimuli (8). These findings are particularly relevant, given that the dorsal ACC is believed to play an important role in attention and executive function, while the rostral ACC is implicated in the assessment and regulation of emotional information, especially in situations that require cognitive control in the presence of high emotional input (16, 27, 28). Moreover, these results are in line with the finding of abnormalities in the ACC among both patients with BPD as well as individuals who engage in non-suicidal self-injury (2, 5–7). As neurobiological research on DBT for BPD is in its infancy, future research replicating these single-study neurobiological findings is clearly warranted to further implicate changes in specific brain regions after DBT treatment.

With regards to the anterior insula, while two studies found reduced activation in the anterior insula after DBT treatment among patients with BPD (17, 21), one study found a significant *increase* in activation in the anterior insula (22). One study also found that reduced activity in the anterior insula was significantly correlated with BPD symptom improvement (17). However, another study found no significant relationship between activation and deactivation of the left anterior insula and medial occipital lobe with BPD symptoms (22). Future research clarifying the association between activation/deactivation of the anterior insula and DBT treatment outcomes are warranted, given the role of the anterior insula in interoceptive awareness and conscious evaluation of bodily states (29–31).

Three separate studies examined the impact of DBT treatment on the inferior frontal gyrus (16, 19, 21). These studies found that after DBT treatment, patients with BPD experienced an increase in gray matter in the inferior frontal gyrus (16), decreased activity in response to arousing stimuli (21), and increased activity in response to inhibitory control (19). These findings suggest that both the structure and function of the inferior frontal gyrus may be significantly altered after DBT treatment among patients with BPD (16, 19, 21).

In addition to specific brain structure and function, it is worth noting here that DBT may lead to alterations in neurotrophin methylation (32). One study found that after a 4-week intensive DBT program, BDNF methylation significantly increased across all BPD patients (32). The authors noted that DBT non-responders accounted for the majority of this increase, whereas DBT responders actually showed a *decrease* in BDNF methylation over time (32). The patients with the best response achieved the level of BDNF methylation found in healthy control patients (32). Thus, it appears that BDNF methylation may be associated with DBT treatment response among BPD patients (32).

### Neural Correlates of Other Treatments/Populations

In addition to the impact of DBT on neural changes among patients with BPD, there have been several other studies that have examined neural changes across various types of treatments and clinical populations. The finding that DBT treatment directly impacts changes in the posterior cingulate cortex, hippocampus, and other frontal areas that are similar to previously described changes in neuroimaging following psychotherapy across a variety of mental disorders, including interpersonal therapy for major depression (33) as well as cognitive-behavioral therapy for major depression (34), specific phobia (35), and social phobia (21, 36).

Furthermore, there have been several studies that have specifically examined the neural impact of other types treatments among individuals with BPD. One study among patients with BPD with affective instability found that BPD patients showed decreased activity in the left orbitofrontal cortex and increased activation of the bilateral insula compared to healthy controls when attempting to downregulate their negative emotional responses using cognitive reappraisal after viewing negative pictures (37). Another study found that after using a distancing technique, in which the subject had to increase the sense of objective distance, BPD patients showed decreased brain responses in the dorsal ACC and inferior parietal lobe, and less deactivation in the amygdala compared to healthy control patients (38, 39). However, one study found no significant difference in the activation of the ACC among trauma-exposed women with BPD compared with healthy controls after using a cognitive reappraisal technique to down-regulate negative emotions after reading negative scripts designed to increase negative emotions (39).

Several studies have focused on the treatment strategy known as neurofeedback, which involves instructing participants to down-regulate their emotional response after viewing neurofeedback pertaining to activation of a specific brain region (40, 41). One study that involved BPD patients receiving a 4-day training on how to monitor amygdala blood oxygenation level neurofeedback among BPD patients found that although amygdala neurofeedback was associated with decreased right amygdala activation after viewing aversive pictures, this decreased activation did not persist when participants were instructed to apply down-regulation without receiving any feedback (40). A more recent study found that 4 days of amygdala neurofeedback training was associated with both amygdala deactivation as well as a decrease in BPD symptoms and a decrease in emotion-modulated startle to negative pictures among BPD patients (41). Future research on the positive impact of brief amygdala neurofeedback on the neural functioning of individuals with BPD may offer a unique alternative to prolonged DBT treatment (40, 41).

Finally, there have been a handful of studies examining the neural impact of psychodynamic therapy among individuals with BPD (42, 43). One study of BPD patients found increased dorsal prefrontal (i.e., dorsal anterior cingulate, dorsolateral prefrontal, and frontopolar cortices) activation, and relative decreased ventrolateral prefrontal cortex and hippocampal activation following psychodynamic treatment (42). This study also found that improvement in constraint correlated positively with relative increased left dorsal anterior cingulate cortex activation (42). As well, improvement in affective lability correlated positively with left posterior-medial orbitofrontal cortex/ventral striatum activation, and negatively with right amygdala/parahippocampal activation (42). Two other studies with small sample sizes of BPD patients (i.e., *n* = 2) found stronger frontal region activation (43) as well as increases in frontal serotonergic transmission compared to non-treated BPD patients (44). Similar to amygdala neurofeedback, future research on the positive impact of psychodynamic therapy on the neural functioning of individuals with BPD may offer a unique alternative to DBT treatment (42–44).

### Biomarkers Predicting DBT Treatment Success

In addition to neural changes *after* DBT treatment among BPD patients, there has also been several lines of evidence on specific neural correlates *prior* to the commencement of DBT that may be associated with improved outcome following treatment (19, 20, 45). One study found that, compared to BPD patients who either dropped out or did not initiate DBT, BPD patients who completed the 6-months of DBT showed less pre-treatment activation during inhibitory control in a region within the left PFC encompassing the inferior and middle frontal gyri, and the superior frontal gyrus, along with less activation in right middle frontal gyrus (19). In contrast, BPD patients who did not complete treatment showed greater pre-treatment activation across a large area encompassing the medial PFC, consisting of superior and middle frontal gyri bilaterally, along with the right inferior frontal gyrus (19). Additionally, a sub-region within the left middle frontal gyrus demonstrated lower activation during response inhibition among patients with BPD who did not complete treatment (19). A follow-up study using the same sample found that BDP patients that reduced their frequency of self-harm the most over treatment displayed lower levels of neural activation in the bilateral dlPFC prior to beginning treatment (20).

A second study found that amygdala and parahippocampus activation during a cognitive reappraisal task after viewing negative pictures, severity measures of BPD psychopathology, and gray matter volume of the amygdala provided best predictive power for identifying DBT treatment non-responders (45). Prior to starting DBT, this study found that treatment non-completers demonstrated greater activation than treatment-completers in the medial PFC and right inferior frontal gyrus (20). One other study found that the presence of the 12-repeat allele on the VNTR polymorphism of SERT was associated with higher adherence to DBT treatment among BPD patients (46).

Moreover, one study of BPD patients undertaking psychodynamic therapy found that post-treatment improvements in constraint were predicted by pre-treatment right dorsal anterior cingulate cortex hypoactivation, while pre-treatment left posterior-medial orbitofrontal cortex/ventral striatum hypoactivation predicted improvements in affective lability (42). One study among BPD patients who underwent 12-weeks of DBT treatment found that while there were no significant differences in DNA methylation of APBA3 and MCF2 differences between patients with BPD and healthy controls, there was a significant correlation between the methylation status of APBA3 and MCF2 and therapy outcome. More specifically, before DBT treatment, both genes were significantly higher methylated in BPD patients who responded to DBT compared to patients with BPD that did not respond to DBT (47).

## Concluding Remarks

It is clear that there is much to learn about both the unique impact of DBT as well as other forms of treatment on individuals with BPD, along with learning more about how baseline genetics and neural patterns of brain functioning may be helpful in predicting treatment success in BPD (2, 19). However, research on the neurobiological changes of DBT as well as biomarker research on DBT treatment success is still in its infancy. Given that the majority of studies use a single pre-post design when administering neuroimaging, future research using multiple scans throughout the course of treatment can help confirm the specific effects of psychotherapy on the brain over-and-above general symptom improvement over time (23). As well, DBT dismantling studies can further elucidate if there are significant neurobiological changes based on each individual DBT module (e.g., mindfulness, emotion regulation, distress tolerance, interpersonal effectiveness) (22). Moreover, future research can help optimize DBT treatments by incorporating neuroimaging biomarkers that could hold promise as therapeutic targets used to track and predict treatment-related improvement among individuals with BPD (2, 19).

## Author Contributions

AI conceived the topic idea and wrote the manuscript. EBL helped supervise the project. All authors were involved in the editing of the manuscript.

## Conflict of Interest

The authors declare that the research was conducted in the absence of any commercial or financial relationships that could be construed as a potential conflict of interest.

## Publisher's Note

All claims expressed in this article are solely those of the authors and do not necessarily represent those of their affiliated organizations, or those of the publisher, the editors and the reviewers. Any product that may be evaluated in this article, or claim that may be made by its manufacturer, is not guaranteed or endorsed by the publisher.
